# Protein Kinase G Iα Inhibits Pressure Overload–Induced Cardiac Remodeling and Is Required for the Cardioprotective Effect of Sildenafil In Vivo

**DOI:** 10.1161/JAHA.112.003731

**Published:** 2012-10-25

**Authors:** Robert M. Blanton, Eiki Takimoto, Angela M. Lane, Mark Aronovitz, Robert Piotrowski, Richard H. Karas, David A. Kass, Michael E. Mendelsohn

**Affiliations:** Molecular Cardiology Research Institute and Division of Cardiology, Tufts Medical Center, Boston, MA (R.M.B., A.M.L., M.A., R.H.K., M.E.M.); Department of Medicine, Tufts Medical Center, Boston, MA (R.P.); Division of Cardiology, Department of Medicine, Johns Hopkins School of Medicine, Baltimore, MD (E.T., D.A.K.)

**Keywords:** heart failure, nitric oxide, protein kinase G, remodeling heart failure, signal transduction

## Abstract

**Background:**

Cyclic GMP (cGMP) signaling attenuates cardiac remodeling, but it is unclear which cGMP effectors mediate these effects and thus might serve as novel therapeutic targets. Therefore, we tested whether the cGMP downstream effector, cGMP-dependent protein kinase G Iα (PKGIα), attenuates pressure overload–induced remodeling in vivo.

**Methods and Results:**

The effect of transaortic constriction (TAC)–induced left ventricular (LV) pressure overload was examined in mice with selective mutations in the PKGIα leucine zipper interaction domain. Compared with wild-type littermate controls, in response to TAC, these Leucine Zipper Mutant (LZM) mice developed significant LV systolic and diastolic dysfunction by 48 hours (n=6 WT sham, 6 WT TAC, 5 LZM sham, 9 LZM TAC). In response to 7-day TAC, the LZM mice developed increased pathologic hypertrophy compared with controls (n=5 WT sham, 4 LZM sham, 8 WT TAC, 11 LZM TAC). In WT mice, but not in LZM mice, phosphodiesterase 5 (PDE5) inhibition with sildenafil (Sil) significantly inhibited TAC-induced cardiac hypertrophy and LV systolic dysfunction in WT mice, but this was abolished in the LZM mice (n=3 WT sham, 4 LZM sham, 3 WT TAC vehicle, 6 LZM TAC vehicle, 4 WT TAC Sil, 6 LZM TAC Sil). And in response to prolonged, 21-day TAC (n=8 WT sham, 7 LZM sham, 21 WT TAC, 15 LZM TAC), the LZM mice developed markedly accelerated mortality and congestive heart failure. TAC induced activation of JNK, which inhibits cardiac remodeling in vivo, in WT, but not in LZM, hearts, identifying a novel signaling pathway activated by PKGIα in the heart in response to LV pressure overload.

**Conclusions:**

These findings reveal direct roles for PKGIα in attenuating pressure overload–induced remodeling in vivo and as a required effector for the cardioprotective effects of sildenafil.

## Introduction

The cyclic GMP (cGMP) intracellular signaling pathway attenuates pathologic cardiac hypertrophy and remodeling in experimental models of heart failure and is upregulated by a number of established and investigational treatments of heart failure.^1–14^ However, the molecules that mediate the cGMP antiremodeling effect remain unknown.^15^ In the heart, cGMP is synthesized by guanylate cyclase (GC), which can be activated at the cell membrane by natriuretic peptides or in the cytosol by nitric oxide (NO) (reviewed in ^16^). Sildenafil, which is under active investigation for the treatment of heart failure, also increases intracellular cGMP by inhibiting its catabolism by phosphodiesterase 5 (PDE5). In the heart, cGMP regulates PDEs and ion channels and activates cGMP-dependent protein kinase G I (PKGI).^17,18^ Identifying which of these cGMP effectors attenuate cardiac remodeling in vivo could therefore reveal important insights into the pathophysiology of heart failure and could identify novel therapeutic targets for the treatment and prevention of this disease. In addition, although natriuretic peptides and nitric oxide synthase can attenuate cardiac remodeling in experimental models of heart failure, they can also activate separate proremodeling pathways under certain conditions,^5,19^ thus making identification of the specific downstream cGMP effectors critical to the design of new therapeutic approaches.

There are 2 isoforms of PKGI, Iα and Iβ, which differ only in their N-terminal leucine zipper (LZ) domains.^15^ In the heart, PKGIα is the predominant isoform.^20^ Currently, however, whether PKGIα attenuates cardiac hypertrophy has not been directly tested in vivo, because animal models lacking PKGI exhibit early lethality, thus precluding study of their cardiac phenotype.^21^ The present study was designed to examine the role of PKGIα in response to LV pressure overload in vivo in order to gain insight into the pathophysiology of cardiac remodeling and heart failure. To this end, we used a novel mouse model, the PKGIα Leucine Zipper Mutant (LZM) mouse, which harbors discrete mutations within the PKGIα LZ domain but maintains normal PKGIα kinase activity.^22^ We tested the response of these LZM mice to transaortic constriction (TAC), a standard experimental model of pressure overload–induced cardiac remodeling and heart failure,^23^ and compared the myocardial signaling pathways activated in WT and LZM hearts after TAC.

## Methods

### Animal Models and Care

PKGIα LZM mice were generated on a C57/BL6 background as described previously.^22^ In all animal studies, the investigators were blinded to the genotype of the mice throughout, including during surgery, in vivo studies, and data analysis. Animal care was in accordance with and approved by the Institutional Animal Care and Use Committee of Tufts University School of Medicine and Tufts Medical Center.

### Transaortic Constriction

TAC^23^ was performed as reported previously.^24^ Briefly, 10- to 14-week-old male mice were anesthetized with 2.5% isoflurane, and body temperature was maintained at 37°C using a closed loop system (Barnant Co, Barrington, IL). To avoid the confounding effects of prolonged systemic hypertension, we performed TAC on 10- to 14-week-old male mice or, in the 48-hour TAC experiment, on young adult male mice (less than 6 months of age). In each experiment all mice were matched for body weight and age. Distinct cohorts of mice were studied in which animals were sacrificed 48 hours, 7 days, or 3 weeks after TAC. In the sildenafil experiments, mice were fed a soft diet (Bioserve) supplemented with sildenafil as described,^1^ at a dose of 200 mg/kg per day,^25^ as previously reported.

### LV In Vivo Hemodynamic Measurements

After anesthetizing mice with 2.5% isofluorane, hemodynamic analyses were performed from the right carotid artery using a fully calibrated, 1.0-Fr catheter (PVR-1045; Millar Instruments, Houston, TX). TAC pressure gradients were also quantified by cannulating the left carotid with a 1.0-Fr microtip pressure transducer (model SPR-1000; Millar Instruments) and measuring aortic pressure distal to the stricture. The TAC gradient was then calculated by subtracting the left carotid pressure from the right carotid systolic pressure, as described.^23^ Hemodynamics were recorded and analyzed with IOX version 1.8.11 software (EMKA Instruments, Falls Church, VA).

### Tissue Histology

Hearts were fixed in end diastole by direct intracardiac injection of KCl. After organ removal, the atria and great vessels were carefully excised, and the RV was dissected from the LV. Organs were fixed in 10% formalin, embedded in paraffin, sectioned into 4-μm samples, and then stained with H&E. Myocyte measurements were performed on sections obtained at the midpapillary muscle level of the LV. For measuring cardiac myocyte cross-sectional area, images at the midpapillary level were acquired at 40× using the SPOT Basic 4.7 program (at least 2 fields per heart). Cardiac myocytes (CMs) with visible central nuclei were traced in cross-section using Image-Pro version 6.2. Equal numbers of CMs were analyzed per group, and statistical analysis was performed on myocyte groups by 1-way ANOVA.

### Transthoracic Echocardiography

For unanesthetized echocardiograms, adult male mice were acclimated to the procedure for 4 training sessions over a 2-day period. On the third day, echocardiography was performed. M Mode and 2-dimensional images were obtained from the short-axis view, as described previously.^24^ Left ventricular end-diastolic and end-systolic diameters (EDDs and ESDs, respectively) and heart rate were measured by averaging values obtained from 5 cardiac cycles. Fractional shortening was calculated using the following standard equation: FS%=([EDD−ESD]/EDD)×100. The investigators were blinded to genotype during data acquisition and analysis. For quantification of TAC gradients, flow velocity across the TAC was measured by Doppler, and the pressure gradient was quantified with the modified Bernouli equation (pressure gradient=4V^2^).

### Cardiac Signaling Studies and Immunoblotting

For generation of cardiac protein lysates, hearts were rapidly excised from mice under 3% isoflurane anesthesia, followed by snap-freezing in liquid nitrogen. Tissue was lysed by dounce homogenization in ice-cold lysis buffer containing 50 mmol/L Tris Cl (pH 7.6), 7 mmol/L MgCl_2_, 2 mmol/L EDTA, 2 mg/mL *N*-dodecyl-B-maltoside, 0.4 mg/mL cholesteryl hemisuccinate, 1 mmol/L PMSF, 1× Protease Inhibitor Cocktail (Calbiochem), and 1× Halt Phosphatase Inhibitor Cocktail (Pierce). After further lysis with 5 passes through a 22-gauge needle, samples were incubated on ice for 1 hour, followed by centrifugation at top speed in a microcentrifuge for 20 minutes. The supernatants were saved, protein concentration was quantified by BCA assay (Pierce), and samples were denatured in Laemlli Sample buffer and then heated at 100°C for 5 minutes. Proteins were resolved by polyacrylamide gel electrophoresis, followed by electrotransfer to Protran nitrocellulose. The membrane was blocked with TBS containing 0.1% Tween and 5% milk powder. Primary antibody incubation (1 hour) in blocking solution was followed by washing (15 minutes, then 5 minutes ×3) and the addition of secondary horseradish peroxidase–conjugated anti-rabbit IgG (1:1000 dilution; GE Bioscience) or anti-mouse IgG (1:1000; GE Bioscience) in blocking solution (1 hour). After washing, membranes were developed on film using ECL (Amersham). For densitometric analysis, immunoblotted bands were quantitated using an Alpha Innotech image analyzer, and the data were plotted using SigmaPlot. Antibodies were raised against the LZM N-terminus (residues 1 to 59), as described previously.^22^ Commercial antibodies were used to PKGIcommon (1:1000 dilution; Assay Design KAP-PK005, rabbit polyclonal), JNK (1:1000), phospho-JNK (1:1000; Cell Signaling), MKK4 (1:200; Cell Signaling) phospho-MKK4 (1:500 Cell Signaling), and PKGIβ (1:500; Santa Cruz).

### Statistics

All data are reported as mean±SEM. Comparisons between 2 groups were performed using the unpaired Student *t* test. For TAC experiments, comparisons were made by 2-way analysis of variance (ANOVA) or, when noted, by 1-way ANOVA, and *P* values shown indicate the effect of genotype on the TAC-stimulated response. Correction for multiple comparisons was made using the Student–Newman–Keuls method unless noted. A *P* value <0.05 was considered statistically significant. Statistics were analyzed using SigmaStat software.

## Results

The generation of the PKGIα LZM mouse has been reported previously.^22^ Briefly, these mice harbor a knock-in mutation in which the first 4 hydrophobic leucine/isoleucine residues of the PKGIα LZ domain have been replaced with alanines to disrupt the coiled-coil tertiary structure of this region, yielding a protein with retained PKGIα kinase activity but abolished binding with proteins whose binding is dependent on the LZ. These knock-in mice have normal Mendelian inheritance, grow normally, and have a normal life span. They display significant systemic hypertension but not until after 6 months of age.^22^

### Baseline PKGI Expression in Hearts of PKGIα Leucine Zipper Mutant Mice

**Figure 1.**
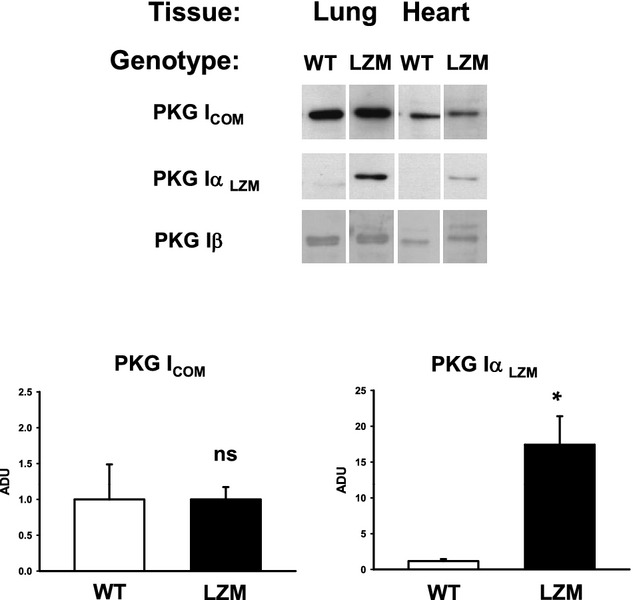
LZM hearts and lungs express LZM-PKGIα mutant protein. Representative immunoblots of heart and lung lysates from WT and LZM mice with antibodies recognizing an epitope common to both isoforms of PKGI (PKG I_COM_), PKGI LZM protein only (PKG Iα_LZM_), or WT PKGIβ only (PKG Iβ). Total PKGI and PKGIβ expression levels were unchanged by the presence of the targeted mutation. Relative protein expression detected by PKG I_COM_ or PKG Iα_LZM_ antibodies is also shown. *P<0.05; n=3 hearts per genotype.

We first examined wild-type and mutant PKG protein expression in hearts from WT and LZM mice. PKGIα LZM mutant protein expression was observed in cardiac tissue of adult LZM mice (Figure 1), but not in the WT littermate control mice. In addition, total PKGI and PKGIβ expression levels were unchanged in the LZM hearts and lungs, compared with those of the WT littermates (Figure 1).

### LZM Left Ventricles Have Impaired Functional Compensation 48 Hours After Pressure Overload

**Figure 2.**
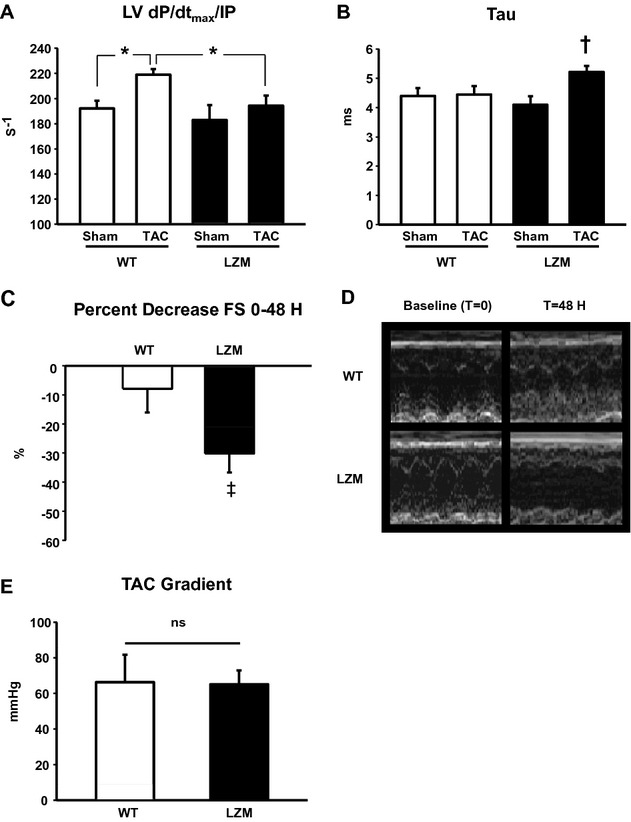
Impaired systolic and diastolic compensation after 48 hours of pressure overload in LZM left ventricles. Summary data of (A) LV Contractility Index (LV dP/dt_max_ normalized to instantaneous pressure, IP), and (B) Tau, obtained from invasive hemodynamic measurements from anesthetized adult male mice (n=6 WT sham, 6 WT TAC, 5 LZM sham, 9 LZM TAC). C, Summary data of percent change in fractional shortening in unanesthetized adult male mice from baseline to 48 hours post-TAC (n=10 WT, 10 LZM). D, Representative echocardiograms at baseline and 48 hours post-TAC. E, Direct measurement of gradient across TAC. *P<0.05; ^†^*P*<0.05 vs WT TAC, LZM sham; ^‡^*P*<0.01 vs WT TAC.

To test directly the role of PKGIα in regulating the cardiac response to LV pressure overload in vivo, we subjected separate cohorts of young adult male LZM or WT littermates to LV pressure overload by TAC for multiple durations: 48 hours, 7 days, and 21 days. Forty-eight hours following TAC, in the WT mice, LV contractility index (CI, dP/dt_max_/instantaneous pressure) increased significantly compared with sham mice (219.0±4.6 s^−1^ in WT TAC versus 192.1±6.2 s^−1^ in WT sham; *P*<0.05) (Figure 2A). However, in LZM mice, CI did not increase significantly with TAC compared with sham mice, and CI was significantly lower than in the WT TAC group (194.2.0±8.1 s^−1^ in LZM TAC, 182.9±12.0 s^−1^ in LZM sham; *P*<0.05, LZM TAC versus WT TAC; *P*=ns, LZM TAC versus LZM sham) (Figure 2A). In WT hearts 48 hours post-TAC, diastolic function measured by the index Tau (the time constant of isovolumic relaxation) did not change significantly compared with sham controls (4.5±0.3 ms WT TAC versus 4.4±0.3 ms WT sham, *P*=ns) (Figure 2B). However, in the LZM mice, diastolic function worsened significantly in the LZM TAC mice compared with WT TAC mice (Tau 5.2±4.2 ms LZM TAC, 4.5±0.3 ms WT TAC, 4.1±0.3 ms LZM sham; *P*<0.05 LZM TAC versus WT TAC; *P*<0.05 LZM TAC versus LZM sham) (Figure 2B). In WT mice, percentage of fractional shortening (FS%) significantly worsened following 48 hours of TAC (−7.9±−8.2% in WT). The relative FS% decrease in LZM hearts 48 hours post-TAC was significantly more severe compared with the WT TAC group (−30.2±−6.6% from *t*=0 to *t*=48 hours post-TAC in LZM; *P*<0.05 versus WT) (Figure 2C).

We also directly measured the degree of LV pressure overload in experimental groups. Forty-eight hours after TAC, the measured TAC gradients did not differ between the WT and LZM mice (WT TAC gradient 66.3±15.3 mm Hg, LZM TAC gradient 66.0±7.8 mm Hg; *P*=ns WT versus LZM) (Figure 2E). Systolic pressures did not differ between genotypes in sham mice or within genotypes of TAC-treated mice (data not shown), further demonstrating that the more severe response of LZM LVs to pressure overload did not arise from differences in afterload experienced by the LV.

### LZM Left Ventricles Develop Increased Hypertrophy and Contractile Dysfunction in Response to 7 Days of Pressure Overload

**Figure 3.**
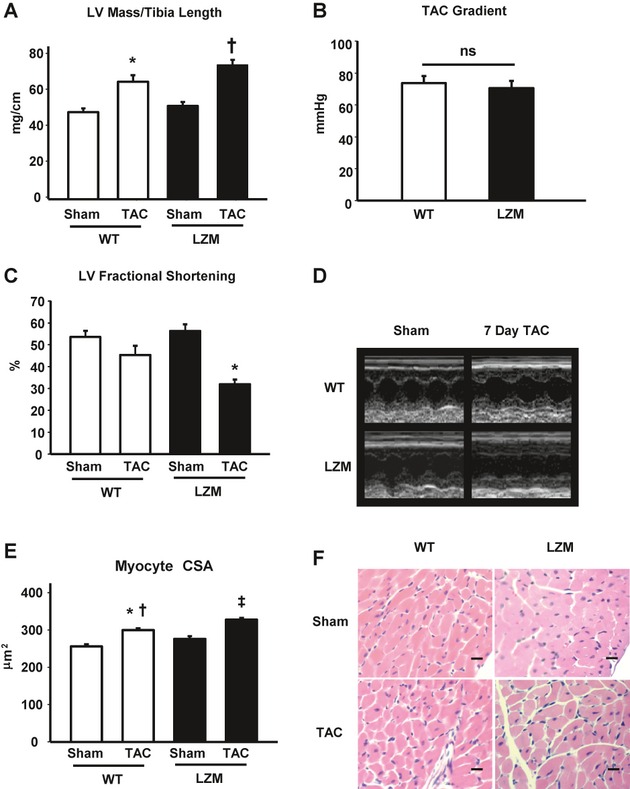
Increased 7-day TAC-induced LV hypertrophy and contractile dysfunction in LZM mice. A, Summary data of LV mass/tibia length in 10- to 14-week-old male mice subjected to 7-day TAC (n=6 WT sham, 5 LZM sham, 11 WT TAC, 14 LZM TAC). *P<0.01 vs WT sham; ^†^*P<*0.05 vs WT TAC, LZM sham. B, Direct measurement of gradient across TAC. C, Summary data of percent fractional shortening (FS) from 10- to 14-week-old male mice subjected to 7-day TAC. *P<0.01 vs WT TAC, WT sham, LZM sham (n*=*5 WT sham, 4 LZM sham, 8 WT TAC, 11 LZM TAC). D, Representative echocardiographic images. E, Quantification of cardiac myocyte cross-sectional area (CSA), n≥200 CMs per group. Cells were analyzed from 3 WT sham, 3 LZM sham, 4 WT TAC, and 5 LZM TAC hearts, >30 myocytes per heart; statistical comparison was with 1-way ANOVA. *P<0.001 vs WT sham; ^†^*P*<0.05 vs LZM sham; ^‡^*P*<0.001 vs LZM sham, WT TAC, WT sham. F, Representative H&E images of LV cross-sections obtained at the midpapillary level.

In a separate cohort of mice, we subjected male 10- to 14-week-old LZM and WT littermate controls to 7 days of LV pressure overload by TAC in order to examine more chronic responses to LV pressure overload. Seven days post-TAC, the LZM mice developed increased LV hypertrophy compared with WT controls (74.7±2.9 mg/cm in LZM TAC versus 65.4±2.2 mg/cm in WT TAC, *P*<0.05) (Figure 3A). Fractional shortening at 7 days, as measured by echocardiography, was significantly lower in LZM TAC mice (31.9±2.3%) compared with the WT TAC group (45.4±4.2%, *P*<0.01 versus LZM TAC) (Figure 3C). LV cardiac myocyte cross-sectional area (CSA) was also larger in LZM TAC mice compared with WT TAC controls (328±5.2 μm^2^ in LZM TAC versus 300±4.5 μm^2^ in WT TAC; *P<*0.001 LZM TAC versus WT TAC, LZM sham, WT sham) (Figure 3E). Seven days after TAC, the measured TAC gradients did not differ between the WT and LZM mice (WT TAC gradient 73.6±4.5 mm Hg, LZM TAC gradient 70.6±4.6 mm Hg; *P*=ns) (Figure 3B). Systolic pressures 7 days postsurgery did not differ between genotypes in sham mice or within genotypes of TAC-treated mice (data not shown), further demonstrating that the more severe response of LZM LVs to pressure overload did not arise from differences in afterload experienced by the LV.

### WT PKGIα Is Required for Sildenafil Antiremodeling Effect in LV Pressure Overload

**Figure 4.**
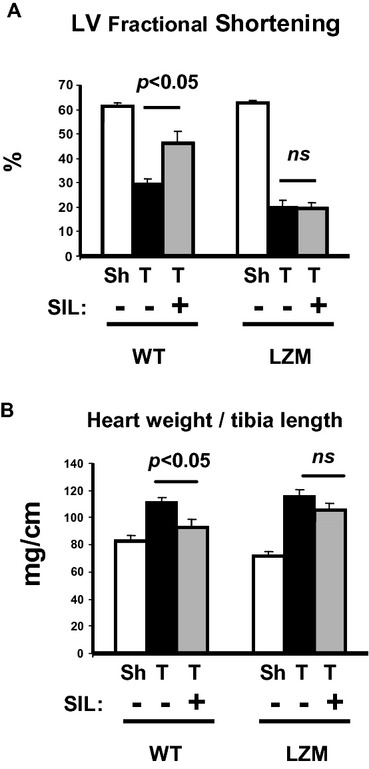
Loss of sildenafil antiremodeling effect in PKGIα–LZM mice. A, Fractional shortening in WT and LZM mice 7 days post-TAC. B, Heart weight/tibia length quantified in WT and LZM mice 7 days post-TAC (n=3 WT sham, 4 LZM sham, 3 WT TAC vehicle, 6 LZM TAC vehicle, 4 WT TAC Sil, 6 LZM TAC Sil). Sh indicates sham; T, TAC. Statistical analysis was performed with 1-way ANOVA, followed by correction for multiple comparisons with the Holm–Sidak method.

The PDE5 inhibitor sildenafil inhibits cGMP catabolism, leading to increased concentrations of intracellular cGMP. Therefore, we tested the hypothesis that PKGIα is required for the previously reported^1^ antiremodeling effect of sildenafil. WT and LZM male mice subjected to 7 days of sham or TAC received the PDE5 inhibitor sildenafil (Sil) 200 mg/kg per day or vehicle (Figure 4A and 4B). In WT controls, TAC-induced decreases in FS% (from 62.1±1.4% in WT shams to 29.7±1.7% in WT TAC vehicle mice) were attenuated by Sil (46.2±5.1% in the WT TAC Sil group; *P*<0.05 Sil versus vehicle), similar to previously published observations.^1^ Sil did not preserve LV FS% in the LZM TAC Sil group, with a reduction in FS% from 62.6±0.6% in LZM shams to 20.2±2.5% in the LZM TAC vehicle group and a similar reduction to 19.3±2.8% in the LZM TAC Sil group (*P*=ns vehicle versus Sil) (Figure 4A). In WT controls, TAC-induced increases in heart weight/tibia length (HW/TL), from 83.0±3.7 mg/cm in shams to 111.6±2.7 mg/cm in vehicle-treated TAC, were also attenuated by Sil (HW/TL 92.7±5.9 in Sil-treated TAC mice; *P*<0.05 Sil versus vehicle; Figure 4B). In LZM mice, however, TAC-induced increases in HW/TL (increased from 71.3±3.3 mg/cm in LZM shams to 115.7±5.2 mg/cm in the LZM TAC vehicle group) were not significantly reduced by Sil (105.5±5.1, *P*=ns LZM TAC Sil versus vehicle) (Figure 4B). These findings provide direct evidence in vivo that normal LZ-mediated targeting of PKGIα is required for the suppression of LV functional decompensation and cardiac remodeling by sildenafil-induced increases in cGMP.

### LZM Mice Have Increased Mortality and More Severe Heart Failure in Response to 21 Days of Pressure Overload

**Figure 5.**
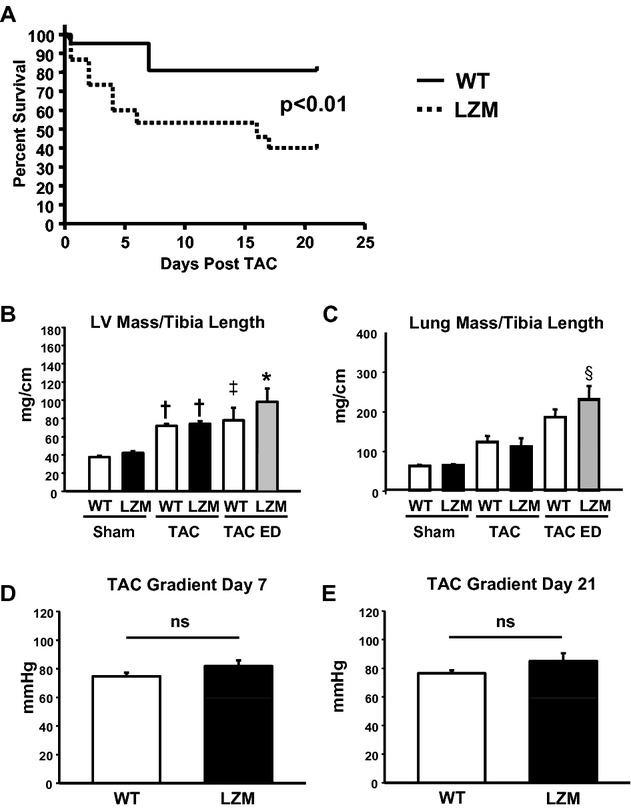
Accelerated LV pressure overload–induced mortality and congestive heart failure in PKGIα LZM mice. A, Kaplan–Meier curve showing markedly accelerated and increased mortality in 10- to 14-week-old male LZM mice subjected to 21-day TAC compared with WT littermate controls. Summary data of (B) LV mass/tibia length and (C) wet lung mass/tibia length in 10- to 14-week-old male mice subjected to 21-day TAC. *P<0.05 vs LZM sham, WT TAC, LZM TAC; ^†^*P*<0.05 vs WT sham, LZM sham; ^‡^*P*<0.05 vs LZM sham; ^§^*P*<0.01 vs WT sham, LZ sham, WT TAC, LZM TAC (n=8 WT sham, 7 LZM sham, 17 WT TAC 21-day survivors [from 21 subjected to TAC], 6 LZM 21-day survivors [from 15 subjected to TAC], 3 WT early death, 6 LZM early death). TAC indicates 21-day survivors; TAC ED, early death induced by TAC. Statistical analysis was performed with 1-way ANOVA followed by correction for multiple comparisons with the Holm–Sidak method. D, Pressure gradient across the TAC determined through noninvasive echocardiographic measurement of aortic flow velocity in a cohort of surviving mice at 7 days (D) and at 21 days (E) post-TAC (n=15 WT TAC survivors at 7 and 21 days, 6 LZM survivors at 7 days, 4 LZM survivors at 21 days).

In a separate cohort of male LZM or WT littermate control mice, 10 to 14 weeks of age, we examined the response to more prolonged, 21-day TAC. In the 21 days following TAC, mortality was markedly increased in the LZM mice compared with the WT mice (60% versus 19%, respectively; *P*<0.01) (Figure 5A. Organs were harvested and weighed at the time of premature death (n=6 measured of 9 deaths in LZM mice and n=3 measured of 4 deaths in WT mice) in mice that died spontaneously and 21 days post-TAC in mice that survived to be studied on day 21. Compared with WT TAC survivors (n=17), LZM mice that died prematurely had a 37% increase in LV mass normalized to tibia length, showing that LZM mice died with exaggerated LV hypertrophy in response to TAC (Figure 5B). The LZM mice that died prematurely also had an 85% increase in the wet lung mass/tibia length ratio compared with the WT TAC survivors, supporting that they developed increased pulmonary congestion (Figure 5C), a consequence of left-sided heart failure.

Calculated noninvasive TAC gradients did not differ significantly between WT and LZM groups at 7 days (WT TAC 74.7±2.5 mm Hg, LZM TAC 81.8±4.0 mm Hg; *P*=ns) (Figure 5D) or 21 days (WT TAC 76.5±2.1 mm Hg, LZM TAC 84.9±5.7 mm Hg; *P*=ns) (Figure 5E). In addition, measured systolic pressure did not differ significantly between genotypes in 21-day shams or between genotypes of 21-day TAC survivors (data not shown), demonstrating that the increased mortality in the LZM TAC mice did not arise from increases in LV afterload compared with WT TAC mice.

### Pressure Overload–Induced JNK Activation Is Selectively Blunted in Hearts of LZM Mice

**Figure 6.**
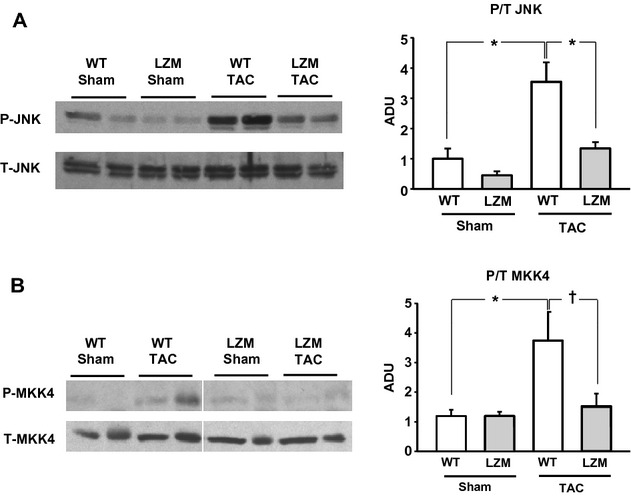
TAC-induced phosphorylation of JNK is reduced in LZM hearts. Representative Western blot and quantitation by densitometry of (A) phosphorylated (P-JNK) and total JNK (T-JNK) from cardiac protein lysates 48 hours after TAC or sham surgery (n=6 hearts per surgical group). B, Phosphorylated (P-MKK4) and total (T-MKK4) from cardiac protein lysates 48 hours after surgery (n=7 WT sham, 6 LZM sham, 4 WT TAC, and 4 LZM TAC hearts per group). *P<0.001; ^†^*P*<0.01.

Because increases in LV dysfunction, heart failure, and total mortality between WT and LZM TAC mice developed by 48 hours post-TAC, we hypothesized that these changes resulted from derangements in early stress-activated myocardial signaling pathways. Therefore, we explored the activation of multiple hypertrophic and remodeling signaling pathways in cardiac lysates from mice exposed to TAC for 48 hours. We initially tested pathways previously implicated as being regulated by PKG in the heart or the CM.^1,25–27^ We therefore examined myocardial JNK phosphorylation, which is regulated by PKGI in the cultured CM^26^ and has also been demonstrated by others to mediate early LV compensation and inhibit hypertrophy in response to pressure overload in vivo.^28,29^ In WT hearts, JNK activation (phosphorylation) increased 3.5-fold, from 1.0±0.3 arbitrary densitometric units (ADUs) in WT sham hearts to 3.5±0.6 ADUs in WT 48-hour TAC hearts (*P*<0.001). However, this increase in JNK activation was not observed in LZM hearts post-TAC (0.5±0.1 ADUs in LZM sham hearts versus 1.3±0.2 ADUs in LZM 48-hour TAC hearts, *P*=NS LZM sham versus LZM TAC; *P*<0.001 LZM TAC versus WT TAC) (Figure 6A). The upstream JNK activator and antihypertrophic MKK4 was also phosphorylated after TAC in WT hearts, from 1.2±0.2 ADUs in WT sham hearts to 3.7±1.0 ADUs in WT TAC hearts (*P*<0.001). Again, this increase in MKK4 activation was not observed in LZM hearts post-TAC (1.2±0.2 ADUs in LZM sham hearts to 1.5±0.4 ADUs in LZM TAC hearts; *P*<0.01 LZM TAC versus WT TAC; *P*=NS LZM sham versus LZM TAC) (Figure 6B). This finding, that TAC induced increased JNK phosphorylation in the WT hearts but not in the LZM hearts, implicates the JNK pathway in the phenotypic changes observed in the LZM mice in response to TAC. The activities of a number of other pathways previously implicated as being regulated by cGMP and PKGI, including calmodulin kinase II, calcineurin, ERK, and AKT, did not differ at 48 hours in LZM TAC hearts compared with WT TAC hearts (data not shown), a time at which LZM mortality and cardiac dysfunction in response to TAC were already increased (Figures 2 and 5).

## Discussion

The present study directly tested the hypothesis that PKGIα attenuates cardiac remodeling in vivo, by examining the effect of genetic disruption of the PKGIα leucine zipper interaction domain on the response to LV pressure overload. We observed in PKGIα-LZM mice subjected to TAC: LV functional decompensation, increased pathologic cardiac hypertrophy, attenuation of the therapeutic effect of sildenafil, and striking congestive heart failure mortality in the setting of chronic pressure overload. Taken together, these findings represent the first direct demonstration that PKGIα normally functions as an antiremodeling molecule in response to LV pressure overload. We also interpret these findings to support further investigation of PKGIα and its downstream phosphorylation targets as therapeutic targets in congestive heart failure.

We performed TAC on LZM mice for multiple durations in order to gain insight into the in vivo role of PKGIα throughout the cardiac remodeling response. As early as 48 hours after TAC, we observed normal early compensation in the LVs of WT mice, but found that LZM LVs had already developed systolic and diastolic dysfunction. These observations support that PKGIα normally functions early in the setting of cardiac pressure overload to attenuate cardiac remodeling and to preserve LV compensation. Others have demonstrated that cGMP levels also increase rapidly in the heart after TAC,^1^ further supporting that cGMP-PKGI signaling plays an early and critical role in attenuating cardiac dysfunction after pressure overload. We also detected abnormalities in Tau, the time constant of diastolic relaxation, in the LZM mice at 48 hours post-TAC, whereas Tau remained preserved in WT controls. PKGI has been proposed to regulate diastolic function in the heart, based on in vitro evidence showing differential regulation of PKGI phosphorylation targets both in failing human hearts^30^ and in experimental models of diastolic heart failure.^31,32^ Our findings, however, demonstrate for the first time in vivo that normal PKGIα is required to maintain LV diastolic function after TAC.

In response to intermediate-duration, 7-day LV pressure overload, we again observed worsening systolic function in LZM mice, but also observed increased LV hypertrophy at the gross and CM level compared with WT controls. These findings support that normal PKGIα not only promotes the initial LV compensation to pressure overload, but also limits pathologic hypertrophy in vivo. Furthermore, we observed in the present study that sildenafil ameliorates cardiac hypertrophy and LV contractile dysfunction in TAC-treated WT hearts, as reported previously,^1^ but now we have also shown that sildenafil fails to do so in LZM hearts subjected to TAC in the same way. Our results therefore identify for the first time that PKGIα is an in vivo mediator of the sildenafil effect in the pressure-overloaded heart. Data from both experimental models of heart failure^1,33^ and from small human clinical trials^34^ demonstrated that sildenafil attenuates LV cardiac hypertrophy and remodeling. As a PDE5 inhibitor, sildenafil increases intracardiac cGMP by inhibiting its catabolism. However, the extent to which PKGIα, a downstream cGMP effector, is responsible for the antihypertrophic effects of increased cGMP had not been tested previously. Now that sildenafil is under active investigation in human heart failure clinical trials^34–39^ and in an ongoing NIH multicenter trial in diastolic heart failure (RELAX study; http://clinicaltrials.gov/ct2/show/NCT00763867), our finding that sildenafil-induced attenuation of remodeling requires intact PKGIα may be especially relevant to our understanding and treatment of human heart failure.

Finally, in our prolonged, 21-day TAC experiments, we observed striking accelerated mortality and increased lung mass/tibia length in the LZM mice, consistent with increased TAC-induced congestive heart failure in the LZM mice. Therefore, these findings also reveal a critical role of PKGIα in inhibiting the development of the clinical syndrome of heart failure in response to LV pressure overload.

Taken together, these findings provide the first direct evidence for the requirement of PKGIα in the inhibition of pressure overload–induced LV dysfunction in vivo. Previous studies of PKGIα phosphorylation targets have only indirectly suggested a role of PKGIα in regulating the LV response to pressure overload. For example, mice with whole-body deletion of the PKGIα kinase target RGS2 demonstrated accelerated LV hypertrophy and mortality in response to TAC and failed to respond to the antiremodeling effect of sildenafil,^25^ thus indirectly implicating PKGIα as regulating this effect. However, our findings in the LZM mice differ from other published in vivo models of PKGI modulation, such as conventional knockout models or pharmacologic studies. For example, mice with full-body deletion of both PKGI isoforms die before adulthood, precluding complete analysis of the cardiac phenotype. And, PKGI “β-rescue” mice with whole-body deletion of PKGI, in which the Iβ isoform was selectively reexpressed only in smooth muscle tissue, were reported to have no differences in cardiac hypertrophy in response to isopreterenol or to TAC, at a single duration after surgery.^40^ Conversely, pharmacologic approaches that limit cardiac remodeling, such as PDE5 inhibition,^1^ activate PKGI indirectly but lack specificity for PKGI activation, making interpretation of the specific role of PKGI difficult. Therefore, we circumvented the above limitations by employing the LZM “knock-in” model in which PKGIα kinase activity is maintained, but only the PKGIα leucine zipper domain is mutated.^22^ Importantly, the LZM knock-in model likely disrupts only the LZ-mediated subset of total PKGI downstream signaling, which may further explain the differences between the phenotypes and the signaling changes observed in the LZM mice compared with conventional knockout approaches or with pharmacological studies.

We interpret these findings to support further investigation of PKGIα and its LZ-mediated effectors in the heart as therapeutic targets in the treatment and prevention of cardiac hypertrophy and heart failure. Identifying downstream cGMP targets that attenuate hypertrophy and remodeling is potentially useful, because upstream activators of cGMP production in the heart can also have proremodeling effects.^5,19^ Therefore, targeting PKGIα, a specific downstream NO-cGMP effector, may prove more effective in circumventing the proremodeling effects of certain upstream cGMP activators. Furthermore, in addition to sildenafil, other cGMP-PKG activating agents are under investigation in heart failure, such as the GC activators cinaciguat and nesiritide.^41,42^ To date, most clinical trials of these agents have focused on their rapid PKGI-mediated vasodilating effects in the setting of acute decompensated heart failure. However, our findings in the present study support extending further clinical investigation of PKGI-activating compounds to the treatment and prevention of chronic cardiac remodeling and congestive heart failure.

Our findings also provide the first evidence in vivo that PKGIα controls antihypertrophic JNK and MKK4 activation in the heart in response to pressure overload. Numerous in vivo studies have established the JNK pathway as an important repressor of cardiac hypertrophy and remodeling. For example, mice with cardiac myocyte-restricted expression of dominant-negative JNK, as well as JNK whole-body knockouts, develop increased hypertrophy after TAC compared with WT controls.^29^ And a number of upstream JNK activators, including MKK4^43^ as well as MKK7,^44^ CDC42,^45^ and MEKK1,^46^ inhibit TAC-induced cardiac remodeling in vivo through JNK-dependent mechanisms. Interestingly, PKGI activates JNK activity in cultured CMs, where it mediates CM survival.^26,47^ Our findings therefore support a novel signaling pathway through which PKGIα preserves LV function and represses hypertrophy and remodeling in response to TAC via downstream activation of JNK. Ongoing studies in our laboratory are exploring the molecular targets through which PKGIα LZ-mediated interactions regulate JNK activation in the heart.

A potential limitation of our study is the possibility that adult-onset hypertension and vascular abnormalities in the LZM mice might confound interpretation of their markedly abnormal response to pressure overload. This is very unlikely, however, because in each of our TAC experiments, both the LV TAC gradients of more than 60 mm Hg and the resultant LV systolic pressures did not differ between WT and LZM TAC mice. Therefore, this demonstrates that the abnormal LZM LV response to TAC does not arise simply from increased pressure overload. In addition, the sham treated mice also had equal basal blood pressures between genotypes, reflecting that we experimented on young mice before the development of detectable hypertension.^22^ The increased LV dysfunction, hypertrophy, and mortality in LZM mice in response to equivalent pressure overload compared with WT strongly supports a cardiac-specific role of PKGIα in inhibiting pressure overload–induced cardiac remodeling in vivo.

Another limitation is that this study did not explore the specific cardiac cell types through which PKGIα antagonizes the pathologic response to pressure overload. However, one advantage of the whole-body approach is that it allowed us to address the clinically relevant questions of whether PKGIα globally inhibits cardiac remodeling and mediates the beneficial effect of sildenafil in the heart. This supports the translational rationale of investigating PKGIα as a novel therapeutic target. Prior studies have already shown that in vitro activation of PKGI or transfection of PKGI into cultured cardiac myocytes inhibits neurohormone-induced cellular hypertrophy,^27,48,49^ supporting an important CM-specific role of PKGI. In vivo models of CM-specific PKGIα deletion are therefore currently under development in our laboratory, and have the potential to clarify the specific cardiac cell types responsible for the PKGIα effect.

In summary, the present study demonstrates for the first time that PKGIα attenuates early LV decompensation and remodeling in response to pressure overload in vivo. Furthermore, our work demonstrates that a functional PKGIα leucine zipper is required both for the cardioprotective effect of sildenafil and for activation of myocardial JNK signaling in response to LV pressure overload. These studies support new therapeutic approaches of activating PKGIα or its downstream myocardial targets in the treatment of humans with pressure overload–induced heart failure.
